# DNAM-1-chimeric receptor-engineered NK cells, combined with Nutlin-3a, more effectively fight neuroblastoma cells *in vitro*: a proof-of-concept study

**DOI:** 10.3389/fimmu.2022.886319

**Published:** 2022-07-28

**Authors:** Chiara Focaccetti, Monica Benvenuto, Chiara Pighi, Alessandra Vitelli, Federico Napolitano, Nicola Cotugno, Doriana Fruci, Paolo Palma, Paolo Rossi, Roberto Bei, Loredana Cifaldi

**Affiliations:** ^1^ Department of Clinical Sciences and Translational Medicine, University of Rome “Tor Vergata”, Rome, Italy; ^2^ Saint Camillus International University of Health and Medical Sciences, Rome, Italy; ^3^ Research Unit of Clinical Immunology and Vaccinology, Dipartimento Pediatrico Universitario Ospedaliero (DPUO), Ospedale Pediatrico Bambino Gesù, Istituto di Ricovero e Cura a Carattere Scientifico (IRCCS), Rome, Italy; ^4^ ReiThera Srl, Rome, Italy; ^5^ Chair of Pediatrics, Department of Systems Medicine, University of Rome “Tor Vergata”, Rome, Italy; ^6^ Department of Paediatric Haematology/Oncology and of Cell and Gene Therapy, Ospedale Pediatrico Bambino Gesù, Istituto di Ricovero e Cura a Carattere Scientifico (IRCCS), Rome, Italy; ^7^ Academic Department of Pediatrics (DPUO), Ospedale Pediatrico Bambino Gesù, Istituto di Ricovero e Cura a Carattere Scientifico (IRCCS), Rome, Italy

**Keywords:** NK cells, immunotherapy combined therapy, activating receptor, chimeric receptor, adoptive transfer of NK and CAR-NK cells, immunomodulation

## Abstract

Adoptive transfer of engineered NK cells, one of clinical approaches to fight cancer, is gaining great interest in the last decade. However, the development of new strategies is needed to improve clinical efficacy and safety of NK cell-based immunotherapy. NK cell-mediated recognition and lysis of tumor cells are strictly dependent on the expression of ligands for NK cell-activating receptors NKG2D and DNAM-1 on tumor cells. Of note, the PVR/CD155 and Nectin-2/CD112 ligands for DNAM-1 are expressed primarily on solid tumor cells and poorly expressed in normal tissue cells. Here, we generated human NK cells expressing either the full length DNAM-1 receptor or three different DNAM-1-based chimeric receptor that provide the expression of DNAM-1 fused to a costimulatory molecule such as 2B4 and CD3ζ chain. Upon transfection into primary human NK cells isolated from healthy donors, we evaluated the surface expression of DNAM-1 and, as a functional readout, we assessed the extent of degranulation, cytotoxicity and the production of IFNγ and TNFα in response to human leukemic K562 cell line. In addition, we explored the effect of Nutlin-3a, a MDM2-targeting drug able of restoring p53 functions and known to have an immunomodulatory effect, on the degranulation of DNAM-1-engineered NK cells in response to human neuroblastoma (NB) LA-N-5 and SMS-KCNR cell lines. By comparing NK cells transfected with four different plasmid vectors and through blocking experiments, DNAM-1-CD3ζ-engineered NK cells showed the strongest response. Furthermore, both LA-N-5 and SMS-KCNR cells pretreated with Nutlin-3a were significantly more susceptible to DNAM-1-engineered NK cells than NK cells transfected with the empty vector. Our results provide a proof-of-concept suggesting that the combined use of DNAM-1-chimeric receptor-engineered NK cells and Nutlin-3a may represent a novel therapeutic approach for the treatment of solid tumors, such as NB, carrying dysfunctional p53.

## Introduction

NK cells are cytotoxic lymphocytes that participate in innate immune responses and recognize virus-infected and transformed cells without prior specific sensitization or fine specificity. Recognition by NK cells of tumor or infected cells is mediated by specific activating receptors (1) that include NKG2D and the accessory molecule DNAX (DNAM-1, CD226) (2). Ligands for the NKG2D receptor are cell stress-inducible molecules such as MICA, MICB (3), and a group of ULBPs (4), whereas ligands for the DNAM-1 receptor are Nectin-2 (CD112) and the poliovirus receptor (PVR, CD155 or nectin-like molecule) (5).

Ligands for the DNAM-1 receptor are expressed at high levels in response to cellular stress (6) in many tumor cell types, especially in solid tumors of epithelial and neuronal origin (7–11), and in virus-infected cells (2, 12), including those infected by SARS-CoV-2 (13). The expression of these ligands can vary, determining the extent to which tumor or infected cells are able to evade the NK cell-mediated immune response (2, 8, 14).

Due to the reduced expression of NK cell-activating receptors in patients with hematological (15–17) and solid tumors (18–20), and the poor infiltration and impaired functions of NK cells in tumor microenvironment (TME) (21), multiple strategies have been adopted to enhance NK cell-mediated anticancer functions. Several approaches aimed at increasing the expression for NK cell-activating receptors (22–24) and their ligands (25, 26), or suppressing NK cell-inhibitory receptors (27–29) are emerging in preclinical studies and several clinical trials (ClinicalTrials.gov and **Supplementary Table S1**). However, if advanced results have been obtained for hematopoietic tumors, in the context of solid tumors many efforts are still needed. Furthermore, adoptive transfer of *extra vivo* expanded and activated NK cells in autologous and allogeneic settings, in combination with monoclonal antibodies (mAbs) recognizing immune checkpoint molecules (30, 31), activating cytokines and immunomodulatory drugs (25, 32) or engineered for Chimeric Antigen Receptors (CARs) (33), emerges as one of the first-line anti-cancer cell immunotherapy strategies with an increasing number of therapeutic clinical successes [(34–36), ClinicalTrial.gov and **Supplementary Table S1**].

The effective activation of DNAM-1 ensures a proper signal to predispose NK cells to induce target cell lysis through cytotoxic granule exocytosis and cytokine production (37). For this reason and based on our previous studies (2, 25, 26, 38–41), here we engineered primary human NK cells to express the DNAM-1-chimeric receptor and explored their ability to recognize *in vitro* target cells including K562 and the LA-N-5 and SMS-KCNR NB cell lines. Human DNAM-1 is a type I transmembrane glycoprotein of ~65 kiloDalton (kDa) containing two Ig-like domains; it is composed of an 18 amino acid (aa) leader sequence, an extracellular domain of 230 aa with two Ig-like C2-set domains, a transmembrane domain of 28 aa and a cytoplasmic region of 60 aa containing two residues (Tyr322 and Ser329) involved in DNAM-1-ligand mediated signal transduction. To explore the efficacy of DNAM-1-chimeric receptor-engineered NK cells, we designed plasmid vectors containing the sequence expressing for DNAM-1 in frame with that for costimulatory molecules such as CD3ζ and 2B4. CD3ζ is a signal-transducing molecule that contains 3 immunoreceptor tyrosine-based activation motifs (ITAMs) and is linked to several activating receptors expressed on the surface of NK cells (42, 43). It provides ITAMs for phosphorylation and activation of T cells expressing CARs, often referred to as first-generation CARs. 2B4 is a member of the CD2 family and recruits SAP and Fyn through cytoplasmic tyrosine motifs. The costimulatory sequence of CD3ζ is an intra-cytoplasmic domain of 112 aa, while the intra-cytoplasmic domain of 2B4 is of 119 aa. We generated four constructs containing: i) the full-length (FL) DNAM-1 sequence, ii) the FL-DNAM-1 sequence in frame with the CD3ζ (52-164 aa) sequence, iii) the DNAM-1 (1-275 aa) sequence, missing the Tyr322 and Ser329 residues, in frame with the CD3ζ (52-164 aa) sequence, iv) the FL-DNAM-1 sequence in frame with both 2B4 (251-370 aa) and CD3ζ (52-164 aa) sequences.

In addition, the potential of NK cells engineered with DNAM-1-based constructs to recognize target cells was evaluated in combination with Nutlin-3a, a small-molecule known to antagonize MDM2, thereby restoring p53 function (44) and, as we previously reported (26), having a strong immunomodulatory function in NB cells. The increased susceptibility of the NB LA-N-5 and SMS-KCNR cell lines after *in vitro* treatment with Nutlin-3a to DNAM-1-engineered NK cells provides a proof-of-concept to design an innovative immunotherapeutic protocol to be adopted for a novel NK cell-based clinical approach to treat solid tumors with dysfunctional p53.

## Materials and methods

### NB cell lines, NK cells and reagents

The following human cell lines were used in this study: human erythro-leukemia cell line K562 (ATCC); NB cell line LA-N-5 (the Leibniz-Institute DMSZ), NB cell line SMS-KCNR (Children’s Oncology Group Cell Culture). The cell lines were characterized by i) authentication by PCR-single-locus-technology (Eurofins-Genomic, Ebersberg, Germany) according to the instructions of the manufacturer, ii) array CGH and iii) routinely tested to confirm the absence of Mycoplasma by Mycoplasma Detection kit (Venor-GeM Advance). Cells were cultured in RPMI 1640 medium supplemented with 10% FBS (Thermo Fisher Scientific), 2 mM glutamine, 100 mg/ml penicillin and 50 mg/ml streptomycin (Euroclone S.p.A.).

Human NK cells were isolated from blood of healthy donors by the RosetteSep NK-cell enrichment mixture method kit (StemCell Technologies) and Ficoll-Paque Plus (Lympholyte Cedarlane) centrifugation. NK cells were routinely checked for CD14^-^ CD19^-^ CD3^-^ CD56^+^ immunophenotype and the expression of activating receptors NKG2D, DNAM-1, NKG2C, the maturation marker CD57, the inhibitory receptor NKG2A, immune checkpoint receptors TIGIT and PD-1 and a panel of inhibitory/activating KIRs such as KIR2DL1/2DS1, KIR2DL2/L3/S2, and KIR3DL1 by flow cytometry. The gate strategy adopted to analyze NK cells is shown in **Supplementary Figure S1**. NK cells with greater than 90% purity and positive for all four inhibitory receptors were suspended in NK MACS medium (Miltenyi Biotec) supplemented with NK MACS Supplement, AB serum and 500 IU/mL of recombinant human IL-2 (PeproTech). NK cells were cultured at 200 x 10^3^ cells/well in 96-well round-bottom plates at 37°C, divided every three days, after a week transferred in cell culture flask T-25 at 2x10^6^ cells/ml, and used up to 20 days after isolation for experiments. NK cells, expanded *in vitro* at a rate of 15 to 20 times at day 20, were transfected with DNAM-1-based vectors to obtain DNAM-1-engineered NK cells (as described below).

LA-N-5 and SMS-KCNR cells were cultured at 37°C in 6-well plates and, at 70% confluence, treated with Nutlin-3a (Cayman Chemical, dissolved in DMSO at 10 mmol/L) at 2 μmol/L or DMSO as control (0.2 μl/ml) for 48 hours. LA-N-5 and SMS-KCNR cells treated with Nutlin-3a or DMSO were tested for the expression of PVR/CD155 and Nectin-2/CD112 (26) and used as target cells in NK cell degranulation assay.

### Antibodies and flow cytometry

The following antibodies for flow cytometry were used: anti-CD107a-APC (H4A3), anti-CD3-AF700 (UCHT1), anti-CD3-PE-CF594 (UCHT1), anti-CD56-PE-Cy7 (B159), anti-CD56-PerCP Cy5.5 (B159), anti-CD57-PE (NK-1), anti-CD45-PE-Cy5 (HI30), anti-NKG2D-BV605 (1D11), anti-NKG2D-PE-CF594 (1D11), anti-CD16-BV510 (3G8), anti-DNAM-1-BV786 (DX11), anti-PD-1-BV421 (MIH4), anti-IFNγ-BV650 (4S.B3), anti-CD14-BV605 (M5E2), anti-CD19-BV650 (SJ25C1) purchased from BD Biosciences; anti-DNAM-1-APC (11A8), anti-NKp46-PE-Cy7 (9E2), anti-TNFα-AF700 (Mab11), anti-CD96-APC (NK92.39) purchased from Biolegend; anti-NKp30-PE (Z25), anti-KIR2DL1/2DS1-PE Cy5.5 (EB6B), anti-KIR2DL2/L3/S2-PE (GL-183) purchased from Beckman Coulter; anti-NKG2A-AF700 (131411), anti-KIR3DL1-APC (DX9) purchased from R&D Systems; anti-NKG2A-FITC (REA110), anti-NKG2C-PE (REA205) purchased from Miltenyi; anti-TIGIT-APC (MBSA43) purchased from eBioscience. All these antibodies were used according to the manufacturers’ protocol. Prior to surface staining, NK cells were pre-stained with Live/Dead™ Fixable Near-IR Dead Cell Stain Kit (Invitrogen). Flow cytometry was performed by using FACSCantoTM II (BD Biosciences) or Cytoflex (Beckman Coulter) and analyzed by FlowJo Software.

### Plasmids, DNAM-1-based constructs and NK cell transfection

Four synthetic genes were designed encoding human full length (FL) DNAM-1 and DNAM-1-based chimeric receptors: 1) FL-DNAM-1; 2) FL-DNAM-1 in frame with CD3ζ (52-164 aa); 3) DNAM-1 (1-275 aa, missing the Tyr322 and Ser329 residues) in frame with CD3ζ (52-164 aa); 4) FL-DNAM-1 in frame with both 2B4 (251-370 aa) and CD3ζ (52-164 aa). DNA sequences encoding the four constructs, optimized for human codon usage, were synthesized by Geneart, Thermofisher. The synthetic genes were then cloned into the expression vector pVJ under the control of the human CMV promoter. Aminoacid sequences of the four chimeric constructs are reported in Supplementary Material and Methods.

Human primary NK cells were *in vitro* expanded by NK MACS medium (Miltenyi Biotec) supplemented with NK MACS supplement and IL-2 and transfected by Amaxa™ P3 Primary Cell 4D-Nucleofector™ X Kit L (Lonza) through Nucleofector^®^ Device (Lonza) with DNAM-1-based constructs (5μg) or the empty vector as control, according to the manufacturing protocol. The pmaxGFP™ Vector provided by the Kit was used to transfect primary NK cells in order to evaluate the transfection efficacy (around 20%, according to the manufacturing protocol). At 24 hours after the transfection, DNAM-1-engineered NK cells were assessed for DNAM-1 surface expression, as well as other receptors, and used for experiments of degranulation, cytotoxicity and cytokine production assays by flow cytometry.

### DNAM-1-engineered NK cell degranulation, cytotoxicity and cytokine production assays

The functions of DNAM-1-engineered NK cells were tested by degranulation, cytotoxicity and cytokine production assays. DNAM-1-engineered NK cells were co-cultured with K562, LA-N-5 or SMS-KCNR target cells at 1:1 ratio for 3 hours, in complete medium in the presence of anti-CD107a (diluted 1:100). During the last 2 hours, GolgiStop (BD Bioscence) was added at 1:500 dilution. Cells were firstly pre-stained with Live/Dead Kit (L/D), stained with anti-CD56, anti-CD16, anti-CD3, anti-CD14, anti-CD19, anti-CD45 and, then, the expression of CD107a was evaluated in the CD14^-^ CD19^-^ CD3^-.^CD56^+^ CD16^+^ CD45^+^ subset by flow cytometry. For the blocking experiments, NK cells were pretreated for 20 min with 25 μg/mL of neutralizing anti-DNAM-1 (DX11, BD-Pharmingen) or anti-NKG2D (149810, R&D Systems) before co-culture with K562 target cells.

The DNAM-1 engineered NK cell cytotoxic activity was tested by a standard 4-hour ^51^Cr-release assay. K562 cells were labelled with ^51^Cr [Amersham International; 100μCi (3.7 MBq)/1 x 10^6^ cells] and were co-cultured (5 x 10^3^) with DNAM-1 engineered NK cells at different effector-target (E:T) cell ratios, in 96-well plates round bottom in triplicates, and incubated at 37°C. At 4 hours of incubation, 25μL supernatant were removed, and the ^51^Cr release was measured with TopCount NXT beta detector (PerkinElmer Life Sciences). The percentage of specific lysis by counts per minute (cpm) was determined as follows: 100 x (mean cpm experimental release – mean cpm spontaneous release)/(mean cpm total release – mean cpm spontaneous release). Specific lysis was converted to lytic units (L.U.) calculated from the curve of the percentage lysis and defined as the number of NK cells required to produce 20% lysis of 10^6^ target cells during the 4-hour incubation.

IFNγ and TNFα production assays were performed by co-culturing NK cells with K562 target cells at 1:1 ratio, in complete medium at 37°C for 12 hours. After 1 h, Brefeldin A (Sigma-Aldrich) 10 μg/ml was added to the co-culture. Cells were pre-stained with L/D, surface-stained as for the degranulation assay (as described above), fixed with 1% paraformaldehyde, permeabilized with Permeabilizing Solution (BD), stained with anti-IFNγ and -TNFα antibodies and analyzed in the CD14^-^ CD19^-^ CD3^-.^CD56^+^ CD45^+^ subset by flow cytometry.

### Statistical analysis

For all data, statistical significance was evaluated with the non-parametric Mann-Whitney test. Normalized values were analyzed for correlation by the regression analysis using GraphPad software. *P* values not greater than 0.05 were considered to be statistically significant.

## Results

### Enhanced DNAM-1-engineered NK cell degranulation, cytotoxicity and cytokine production against K562 cells

**Figure 1:**
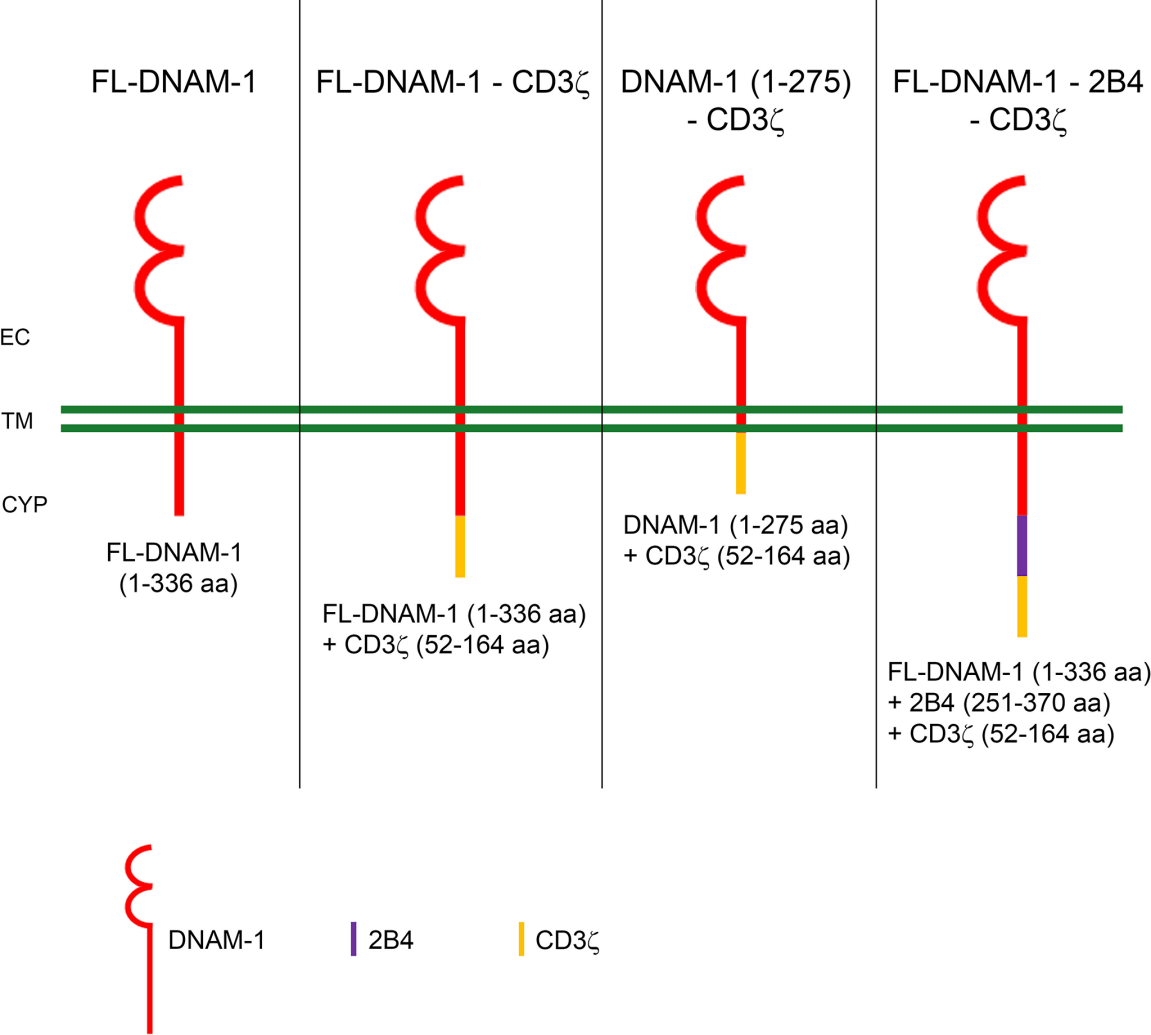
Schematic diagram of DNAM-1-based chimeric receptors. The amino acids (aa) sequences of DNAM-1 (red), CD3ζ (yellow) and 2B4 (blue) are shown below each diagram of DNAM-1-based chimeric receptors. EC, extracellular; TM, transmembrane; CYP, cytoplasm.

**Figure 2:**
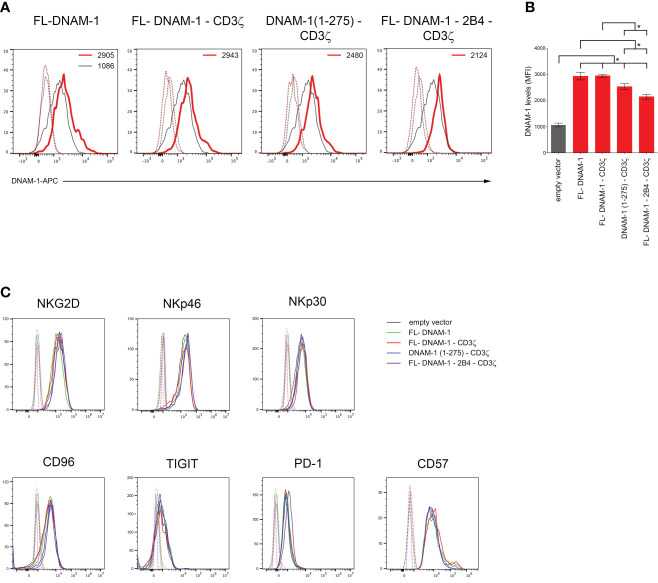
Enhanced surface expression of DNAM-1 receptor on DNAM-1-engineered NK cells. **(A)** Flow cytometry analysis of DNAM-1 surface expression in NK cells transfected with the indicated DNAM-1-based vectors (red) compared to that of NK cells transfected with empty vector (gray). Isotype-matched negative control antibody is displayed as gray and red dashed lines for NK cells transfected with empty vector and each indicated DNAM-1-based vector, respectively. A representative experiment of eight performed in NK cells isolated from independent healthy donors and transfected with DNAM-1-constructs is shown. **(B)** Summary of flow cytometry analyses performed in NK cells isolated by eight independent healthy donors and transfected with DNAM-1-based constructs. MFI, mean fluorescence intensity. Mean ± SD; *p<0.05; *p* value (two-tailed non-parametric Mann-Whitney test). **(C)** Flow cytometry analysis of NKG2D, NKp46, NKp30, CD96, TIGIT, PD-1 and CD57 surface expression in NK cells transfected with the indicated DNAM-1-based vectors. Isotype-matched negative control antibody is displayed as dashed lines for NK cells transfected with empty vector and each indicated DNAM-1-based vector. A representative experiment of seven performed in NK cells isolated from independent healthy donors and transfected with DNAM-1-based vectors is shown.

First, we assessed whether transfection of NK cells with our four DNAM-1-based constructs [FL-DNAM-1, FL-DNAM-1-CD3ζ, DNAM-1 (1-275)-CD3ζ, FL-DNAM-1-2B4-CD3ζ] (**Figure 1**) could affect the surface expression of DNAM-1. NK cells engineered with all four DNAM-1-based constructs showed significantly higher levels of DNAM-1 expression than NK cells transfected with empty vector, as evaluated by flow cytometry (**Figure 2**). Furthermore, both FL-DNAM-1- and FL-DNAM-1-CD3ζ-engineered NK cells showed significantly higher levels of DNAM-1 expression than NK cells engineered with DNAM-1 (1-275)-CD3ζ and FL-DNAM-1-2B4-CD3ζ constructs (**Figure 2B**). In addition, the expression of activating receptors NKG2D, NKp30 (45) and NKp46 (46), the immune checkpoint molecules PD-1 and TIGIT (47), the inhibitory receptor CD96 which, together with TIGIT, is known to compete with DNAM-1 for binding to the same ligands (2), as well as the marker CD57 associated with terminal differentiation of NK cells (48) was unchanged in NK cells engineered with all four DNAM-1-based constructs (**Figure 2C**). These data suggest that in our model i) the intracellular domain of DNAM-1 stabilizes DNAM-1 surface expression; ii) the 2B4 sequence, in frame with DNAM-1 and CD3ζ sequences, partially destabilizes DNAM-1 expression levels; iii) the DNAM-1-based construct transfection does not affect the expression of other receptors.

**Figure 3:**
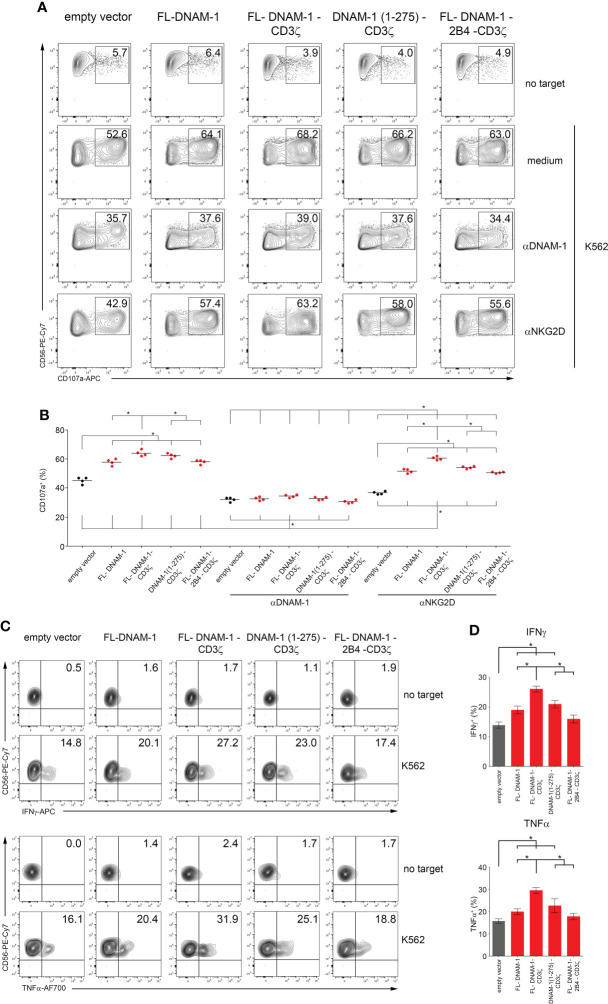
Enhanced degranulation and cytokine production of DNAM-1-engineered NK cells against K562. DNAM-1-engineered NK cells were tested for degranulation and cytokine production assays in response to K562. **(A)** Degranulation of NK cells engineered for the empty vector as control or for the four DNAM-1-based constructs as indicated, in the absence (medium) or presence of neutralizing anti-NKG2D or anti-DNAM-1 antibodies, measured as CD107a expression on co-culture with or without K562. The percentages of CD107a in NK cell subset are indicated in each plot. A representative experiment of four performed in NK cells isolated from independent healthy donors and transfected with DNAM-1-constructs is shown. **(B)** Summary of degranulation of NK cells isolated from four independent healthy donors, transfected with DNAM-1-constructs, in the absence or presence of neutralizing antibodies. Dots correspond to the percentage of CD107a^+^ NK cells from each healthy donor transfected with the indicated DNAM-1-based constructs; horizontal bars indicate the mean; *p<0.05; *p* value (two-tailed nonparametric Mann-Whitney test). **(C)** DNAM-1-engineered NK cells production of IFNγ and TNFα in co-culture with or without K562. The percentages of IFNγ+ and TNFα+ in NK cell subset are indicated in each plot. A representative experiment of four performed in NK cells isolated from independent healthy donors and transfected with DNAM-1-constructs is shown. **(D)** Summary of cytokine production of NK cells isolated from four independent healthy donors and transfected with DNAM-1-based constructs. Mean ± SD; *p<0.05; *p* value (two-tailed non-parametric Mann-Whitney test).

Then, DNAM-1-engineered NK cells were analyzed for degranulation and cytotoxicity assays against K562 cells. As shown in **Figures 3A, B**, **Supplementary Figure S2**, the percentages of CD107a and cytotoxicity were significantly higher in NK cells transfected with all four DNAM-1 constructs than in those transfected with the empty vector. In addition, FL-DNAM-1-CD3ζ-engineered NK cells showed significantly higher CD107a expression and cytotoxicity compared to FL-DNAM-1-, DNAM-1 (1-275)-CD3ζ- and FL-DNAM-1-2B4-CD3ζ-engineered NK cells (**Figures 3B**, **Supplementary Figure S2**). These results suggest that in our model CD3ζ chain conferred a stronger signal to DNAM-1-engineered NK cells than to NK cells transfected with FL-DNAM-1 construct alone, leading to increased degranulation and cytotoxicity in response to a natural target such as K562 cells. However, it was less efficient in the absence of the DNAM-1 intracellular domain or in presence of 2B4 expression.

Furthermore, blocking experiments demonstrated that the chimeric DNAM-1 receptor was involved in the degranulation of NK cells engineered with DNAM-1-based constructs. Indeed, neutralization of DNAM-1 resulted in a drastic reduction of degranulation, with no differences between DNAM-1-engineered NK cells. In contrast, after neutralization of NKG2D, although resulting in a significant reduction of degranulation in all conditions, the differences between the engineered NK cells were comparable to those without neutralizing antibody, as a result of the remaining different contribution of DNAM-1 to the degranulation signal in each condition (**Figures 3A, B**).

In addition, DNAM-1-engineered NK cells were analyzed for the production of cytokines such as IFNγ and TNFα in response to K562 cells, by flow cytometry. As shown in **Figures 3C, D**, the percentage of both IFNγ and TNFα was significantly higher in NK cells transfected with FL-DNAM-1, FL-DNAM-1-CD3ζ and DNAM-1 (1-275)-CD3ζ constructs than in those transfected with the empty vector. In addition, FL-DNAM-1-CD3ζ-engineered NK cells showed significantly higher production of both cytokines IFNγ and TNFα compared to FL-DNAM-1-, DNAM-1 (1-275)-CD3ζ- and FL-DNAM-1-2B4-CD3ζ-engineered NK cells (**Figure 3D**). Therefore, the FL-DNAM-1-CD3ζ construct was shown to be the most effective to confer enhanced degranulation, cytotoxicity and production of both IFNγ and TNFα to NK cells in response to K562 cells.

### Nutlin-3a enhances the susceptibility of LA-N-5 and SMS-KCNR cells to DNAM-1-engineered NK cells

**Figure 4:**
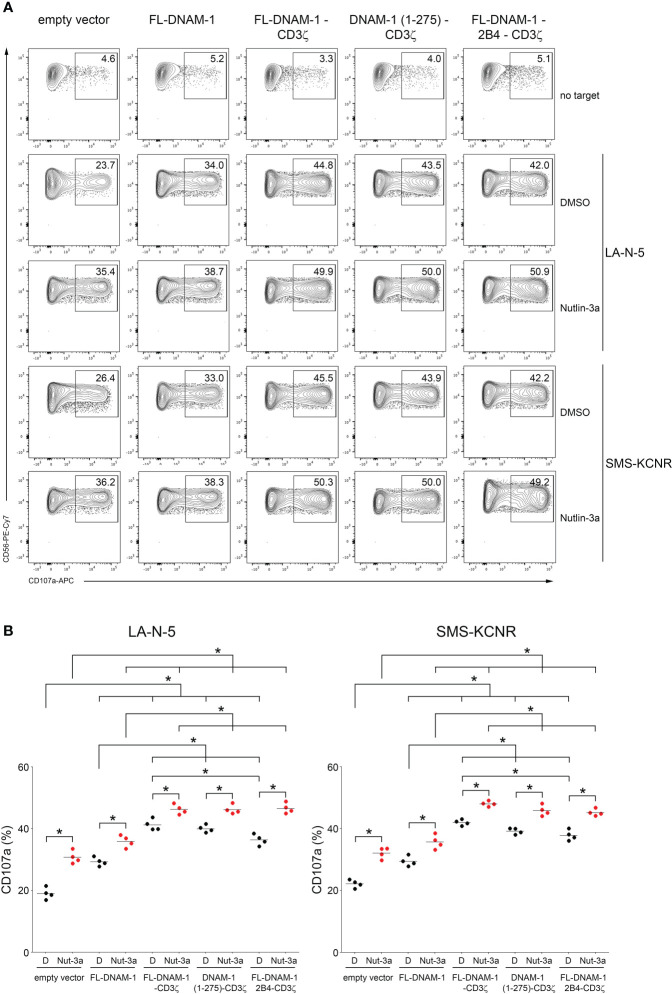
Nutlin-3a boosted the susceptibility of NB LA-N-5 and SMS-KCNR cells to DNAM-1-engineered NK cells. NB LA-N-5 and SMS-KCNR cell lines were left untreated (DMSO) or treated with Nutlin-3a at 2 μmol/L for 48 hours and used as targets for degranulation assay of DNAM-1-engineered NK cells. **(A)** Degranulation of NK cells, measured as CD107a expression upon stimulation with LA-N-5 or SMS-KCNR cells treated with DMSO or Nutlin-3a. The percentages of CD107a^+^ DNAM-1-engineered NK cells are indicated in each plot. A representative experiment of four performed in NK cells isolated from independent healthy donors and transfected with DNAM-1-constructs is shown. **(B)** Summary of degranulation of NK cells isolated from four independent healthy donors and transfected with DNAM-1-constructs, in response to LA-N-5 or SMS-KCNR cells treated with DMSO **(D)** or Nutlin-3a (Nut-3a). Dots correspond to the percentage of CD107a^+^ NK cells from each healthy donor transfected with each indicated DNAM-1-based constructs; horizontal bars indicate the mean; *p<0.05; *p* value (two-tailed non-parametric Mann-Whitney test).

Next, we evaluated the degranulation of DNAM-1-engineered NK cells in response to LA-N-5 and SMS-KCNR NB cells. In addition, we evaluated the combined effect of DNAM-1-engineered NK cells with Nutlin-3a, a drug that antagonizes with MDM2 and, consequently, restores the functions of p53 (44), which is known to act as a transcription factor for genes encoding ligands for NK cell activating receptors (49), including *PVR* for DNAM-1 receptor as we reported (26). To test the effect of Nutlin-3a treatment on the susceptibility of NB cells to DNAM-1-engineered NK cells, LA-N-5 and SMS-KCNR cells were treated with Nutlin-3a or DMSO as a control and used as target cells for DNAM-1-engineered NK cell degranulation assay. As shown in **Figure 4**, the CD107a percentage of NK cells transfected with the four DNAM-1 constructs in response to LA-N-5 or SMS-KCNR cells (treated with DMSO as control) was significantly higher than that of NK cells transfected with empty vector (**Figure 4B**). Furthermore, the degranulation of FL-DNAM-1-CD3ζ-, DNAM-1 (1-275)-CD3ζ− and FL-DNAM-1-2B4-CD3ζ−engineered NK cells in response to LA-N-5 or SMS-KCNR cells treated with DMSO was significantly higher than that of NK cells transfected with FL-DNAM-1 construct. Interestingly, the degranulation of FL-DNAM-1-CD3ζ-engineered NK cells was significantly higher than that of NK cells transfected with FL-DNAM-1-2B4-CD3ζ construct in response to both NB cell lines. These data confirm that CD3ζ chain conferred DNAM-1 constructs with a greater potential to mediate NK cell degranulation in response to NB cell lines such as LA-N-5 and SMS-KCNR cells, which was instead less efficient in the presence of 2B4. Furthermore, Nutlin-3a was able to significantly increase the susceptibility of both LA-N-5 and SMS-KCNR cells to NK cells under all conditions (**Figure 4B**), in agreement with our previous data (26). In response to LA-N-5 or SMS-KCNR cells treated with Nutlin-3a, a significant increase in degranulation was found in NK cells transfected with DNAM-1-based constructs compared to NK cells transfected with empty vector, reflecting the data in response to LA-N-5- or SMS-KCNR treated with DMSO (**Figure 4B**). Interestingly, in response to LA-N-5 or SMS-KCNR cells treated with Nutlin-3a, while a significant increase in degranulation was assessed in NK cells transfected with the three DNAM-1-CD3ζ-based constructs compared to NK cells transfected with the FL-DNAM-1 construct, no differences was revealed between NK cells transfected with the three DNAM-1-CD3ζ-based constructs (**Figure 4B**). These data suggest that Nutlin-3a increased the susceptibility of LA-N-5 and SMS-KCNR cells to NK cells transfected with DNAM-1-CD3ζ-based constructs regardless of the presence of the DNAM-1 intracellular domain or 2B4 sequences. Overall, these data indicated that in our model i) DNAM-1-engineered NK cells had an enhanced ability to recognize NB cell lines such as LA-N-5 and SMS-KCNR, ii) that the CD3ζ sequence in frame with DNAM-1 further enhanced this function, which was maintained by DNAM-1 intracellular domain, but did not require the 2B4 costimulatory sequence. Finally, Nutlin-3a treatment of NB LA-N-5 and SMS-KCNR cells maximized the degranulation of DNAM-1-engineered NK cells, thus providing a proof-of-concept for which its administration, combined with adoptive transfer of DNAM-1-engineered NK cells, may prospectively represent a potential clinical approach.

## Discussion

The treatment of solid tumors, including childhood cancers, still remains a major challenge for oncologists, despite significant advances with multimodal chemotherapy and radiation therapy regimens in combination with several recent immunotherapy approaches.

The efficacy of NK cells has been reported in several tumors as well as in viral infection (21, 50). Of note, the anticancer NK cell-mediated immune response in patients with malignancies is often impaired by the TME cells (e.g. macrophages, myeloid suppressor cells, and stromal/fibroblastic cells), which release inhibitory cytokines/factors (21). Recently, of great interest in the treatment of solid tumors is the adoptive transfer of *ex vivo* expanded and activated NK cells which, due to their peculiar innate characteristics, relatively short lifespan, low risk of hyperproliferation in infused patients, higher safety than infused T cells, and low cost, are an excellent “off-the-shelf” product that could be adopted for a new anticancer therapeutic strategy (51). Furthermore, current good manufacturing practice (GMP) protocols for NK cell adoptive transfer provide for the expansion of NK cells with high activation and low exhaustion status and with greater trafficking and killing performance (34). Thus, the use of adoptive transfer of NK cells appears to be a promising clinical approach to eliminate cancer cells by overcoming the limitations imposed by TME (52, 53).

Currently, several clinical trials indicate that NK cell-based immunotherapy, in combination with cytokines, mAbs, including those recognizing immune checkpoint molecules, is indeed an effective and safe anticancer treatment (21). Furthermore, in the last decade, the use of CARs-armed NK cells, which are thus enhanced in the recognition of specific molecules expressed on the surface of tumor cells, thereby mediating greater tumor eradication, is increasingly emerging in the clinical setting [(54, 55), ClinicalTrial.gov and **Supplementary Table S1**].

Optimizing CAR signal transduction by incorporating additional costimulatory domains is a focal point for improving their anticancer function (56, 57). In addition, identifying anticancer drugs that have additional advantages of immunomodulatory effects, such as induction of ligands for NK cell-activating receptors, in supporting the NK cell- and CAR-NK cell-based immunotherapy still remains challenging (25). Therefore, in the search for more efficient and less toxic therapeutic approaches, new strategies are needed to support and improve NK cell-based immunotherapy of cancer. In this context, the Nutlin-3a-mediated restoration of the p53 function (58), whose abnormality contributes to the severity of various forms of cancer (59), has been widely recognized as an effective and non-toxic therapeutic approach (26, 58, 60).

Here, our results represent a proof-of-concept aimed at designing a novel immunotherapy approach for solid tumors based on the use of DNAM-1-chimeric receptor-engineered NK cells in combination with Nutlin-3a. Our data refer to an *in vitro* exploration on cellular models of NB, a solid tumor known to have dysfunctional p53 (61) and to be able to evade NK cell-mediate immunosurveillance through down-regulation of ligands for NK cell activating-receptors (26, 38, 62, 63). Since the expression of ligands for NKG2D and DNAM-1 receptors is shared in many solid tumors (21, 25), this type of prospective therapeutic approach is expected to be effective not only for NB but also for a broad spectrum of solid tumors with p53 dysfunction, such as colorectal, breast, ovarian, lung and pancreatic cancers (64). Moreover, the DNAM-1 engineered NK cell-based approach should also be effective against infected cells, as the ligands PVR/CD155 and Nectin2/CD112 for DNAM-1 are mainly expressed, not only on solid tumor cells, but also on virus-infected cells (2, 5), including SARS-CoV-2 (13). Interestingly, these ligands are poorly expressed in normal tissues as reported on proteinatlas.org, thus assuming that the use of DNAM-1 engineered-NK cells may be safe.

The prospective success of DNAM-1-engineered NK cells is also supported by the encouraging results regarding to the use of NK cells engineered for different chimeric receptors such as anti-CD19, -PSMA, -5T4, -CD22, -BCMA, -ROBO1, NKG2D, NKG2D-ACE2, as reported in different clinical trials (ClinicalTrial.gov and **Supplementary Table S1**), and anti-GD2, -HER-2, -CS1, -CD20, -EGFR, -PSCA as reported in several preclinical studies (21, 55). Studies concerning the use of NKG2D-chimeric receptor engineered-NK cells, tested for the treatment of leukemia and solid tumors such as colorectal cancer, have shown promising results [(65, 66), ClinicalTrial.gov and **Supplementary Table S1**]. This latter type of chimeric receptor was designed to be expressed in concert with a molecule capable of ensuring signal transduction such as CD3ζ chain and a costimulatory molecule for NKG2D, such as DAP10 (65). As for DNAM-1-chimeric receptors, encouraging data have been reported on engineered T cells against melanoma (67) and the engineered-NK-92 cell line against hepatocellular cancer cells (68) and sarcoma (69).

Here, we generated four different DNAM-1-based expression vectors consisting of the sequence for DNAM-1 in frame with that for the CD3ζ chain and a costimulatory molecule such as 2B4. The choice of co-expressing DNAM-1 with the CD3ζ chain and the costimulatory molecule 2B4 depended on several experimental evidences: i) CD3ζ is a signal transduction molecule that contains three ITAMs associated with different activating receptors expressed on the surface of NK cells (42, 43); it provides ITAMs for intracellular phosphorylation and, thus, activation of CAR-expressing T cells, often referred to as first-generation CARs (70); ii) 2B4 is a member of the CD2 family and recruits SAP and Fyn through tyrosine-based cytoplasmic motifs, thereby mediating its functional relationship with DNAM-1 (71).

We transfected primary human NK cells to obtain DNAM-1-engineered NK cells and performed functional assays against a natural NK cell-target such as K562 cell line and against NB cells such as LA-N-5 and SMS-KCNR cell lines, the latter treated or not with Nutlin-3a. When comparing the target cell recognition and cytokine production abilities of DNAM-1-engineered NK cells transfected with four different DNAM-1-based vectors, FL-DNAM-1-CD3ζ proved to be the best. These data indicated that optimal activation of the DNAM-1 chimeric receptor required the FL-DNAM-1 sequence, including the intra-cytoplasmic sequence. These results were in line with those obtained in the mouse model demonstrating that Tyr319 (Tyr322 in human) is critical for murine DNAM-1-mediated signalling and cytotoxicity (72). Furthermore, optimal activation of the DNAM-1 chimeric receptor required the in-frame sequence CD3ζ (52-164 aa). The cytoplasmic 2B4 domain (251-370 aa) appeared to impair activation, consistent with previous data reporting that anti-CD19-based CAR carrying the 2B4 sequence required both transmembrane and full cytoplasmic 2B4 domains to enhance activation of engineered primary NK cells (73); in contrast, CARs based on anti-CD5 (74) and -GPC3 (68) or -CD19 (75) and -mesothelin (76) carrying the intracytoplasmic 2B4 domain enhanced the activation of engineered NK-92 cell line or primary NK cells, respectively. Thus, both the type of CAR and the nature of NK cells would appear to affect the contribution of the 2B4 sequence to CAR performance differently. Furthermore, blocking experiments by using both anti-NKG2D and anti-DNAM-1 neutralizing antibodies demonstrated the involvement of chimeric DNAM-1 activating receptor in the recognition mediated by DNAM-1-engineered NK cells of K562 cells. Interestingly, Nutlin-3a rendered both LA-N-5 and SMS-KCNR NB cells significantly more susceptible to DNAM-1-engineered NK cells than NK cells transfected with empty vector. This indicated that a synergistic effect occurred between signals triggered by upregulated ligands for both NKG2D and DNAM-1 receptors through Nutlin-3a-mediated immunomodulation (26, 49) and DNAM-1 chimeric receptor. In addition, Nutlin-3a restored the differences between NK cells transfected with DNAM-1-CD3ζ-based constructs evaluated instead in response to DMSO-treated LA-N-5 and SMS-KCNR cells, indicating that it has an immunomodulatory effect that outperforms weaker NK cells transfected with DNAM-1-based constructs, such as DNAM-1 (1-275)-CD3ζ and FL-DNAM-1-2B4-CD3ζ constructs.

Of note, NK cells engineered for DNAM-1-chimeric receptor represent a further advantage in view of the fact that the protocol for *in vitro* expansion and activation of human NK cells, which we adopted, involved the culture of mature, educated and armed cells with the progressive elimination of uneducated hyporesponsive cells (77). On the other hand, the increased expression of the DNAM-1 chimeric-receptor on DNAM-1-engineered NK cells should be an additional advantage over the expression of CD96 and TIGIT, two inhibitory receptors that compete with DNAM-1 for binding to PVR and Nectin-2 or PVR, respectively (2).

However, translating this experimental design to the clinical setting requires many other considerations. Current methods for transfection of primary NK cells for clinical use, such as those with retroviral and lentiviral vectors (78), including pseudotyping with a modified baboon envelop glycoprotein (BaEV-gp) (79), pseudoviral particles (80) or mRNA electroporation (81), to obtain stable, high-efficient, and in large-number DNAM-1-engineered NK cells suitable for the clinical grade, should be applied and, therefore, further investigations are required. In addition, the nontoxic dose of Nutlin-3a used in this study, which showed immunomodulatory effect in both *in vitro* and *in vivo* NB models, as we previously reported (26), or that of its analogues, combined with DNAM-1-engineered NK cells, should be defined in the clinical setting based on the currently applied nontoxic doses of MDM2-targeting drugs reported in several clinical trials for the treatment of different forms of solid tumors (ClinicalTrial.gov and **Supplementary Table S2**).

In conclusion, our results provided a proof-of-concept that the use of FL-DNAM-1-CD3ζ engineered-NK cells in combination with immunomodulatory drugs, such as Nutlin-3a, could represent a novel immunotherapeutic approach to be employed for the treatment of solid tumors with dysfunctional p53. Furthermore, in view of the widely reported involvement of ligands for DNAM-1 in the immune response against cells infected with different types of viruses (2), including SARS-CoV-2 (13), these data suggest to extend the exploration of the use of DNAM-1-engineered NK cells in the context of viral infection.

## Data availability statement

The datasets presented in this study can be found in online repositories. The names of the repository/repositories and accession number(s) can be found in the article/**Supplementary Material**.

## Ethics statement

Ethical review and approval was not required for the study on human participants in accordance with the local legislation and institutional requirements. Written informed consent for participation was not required for this study in accordance with the national legislation and the institutional requirements.

## Author contributions

LC conceived the study and coordinated the project. LC, CF, MB and CP performed the experiments. LC, AV and FN designed DNAM-1-based constructs. LC, AV, NC, DF, PP, PR, and RB discussed the results and provided critical comments. LC wrote the manuscript. All authors critically revised and edited the paper. All authors have read and agreed to the published version of the manuscript.

## Funding

This work was supported by The Italian Ministry of Health (Rome, Italy) 5 X 1000 grant 2020 to LC.

## Acknowledgments

The authors thank Giuseppe Sconocchia, Sara Caratelli and Valeriana Cesarini for kindly providing technical support for the transfection of NK cells using Nucleofector^®^ Device; Paola Vacca for kindly providing critical reagents.

## Conflict of interest

Authors AV and FN are employed by ReiThera Srl.

The remaining authors declare that the research was conducted in the absence of any commercial or financial relationships that could be construed as a potential conflict of interest.

## Publisher’s Note

All claims expressed in this article are solely those of the authors and do not necessarily represent those of their affiliated organizations, or those of the publisher, the editors and the reviewers. Any product that may be evaluated in this article, or claim that may be made by its manufacturer, is not guaranteed or endorsed by the publisher.

## Supplementary material

Supplementary Figure 1 Gating strategy used to analyse NK cell subsets by flow cytometry. *In vitro*-expanded and activated NK cells were checked for the expression of their specific markers at 0 (immediately after isolation from blood of healthy donors), 8, 15 days and before performing functional experiments (15-20 days) of *in vitro* culture in supplemented NK MACS medium. After gating singlet cells (side scatter-A versus side scatter-H), and alive cells (negative for Live/Dead staining), NK cells were identified by gating on CD14^-^ CD19^-^ CD3^-^ subsets to evaluate in CD56^+^ CD16^+^ subset the expression of surface markers, as indicated. NK cells expressing NKG2A, KIR2DL1/2DS1, KIR2DL2/L3/S2 and KIR3DL1 were used for experiments. A representative staining of expanded and activated NK cells at 8 days of culture is shown. The expressions of CD3, CD56 and CD16 in cultured NK cells were compared to those in PBMCs isolated from the same healthy donors. The percentages of NK cells are shown in CD56 vs CD16 dot plots.

Supplementary Figure 2 The cytotoxic activity of DNAM-1-engineered NK cells was evaluated against K562 cells at the indicated effector:target (E:T) ratios by a standard 4-hour ^51^Cr-release assay. A representative experiment out of three performed is shown. The specific lysis from cytotoxic activity in triplicates were converted to L.U. 20% (right panel). The dots in the curves (left panel) and bars in the histogram (right panel) indicate the mean ± SD of triplicates; **p*<0.05; *p* value (two-tailed non-parametric Mann-Whitney test; in the left panel, *1, empty vector versus all four DNAM-1-based vectors; *2, FL-DNAM-1 vectors versus all three DNAM-1-CD3ζ-based vectors.
