# Supplementary figures and images for: DNAM-1-chimeric receptor-engineered NK cells, combined with Nutlin-3a, more effectively fight neuroblastoma cells in vitro: a proof-of-concept study
Source: Front Immunol. 2022 Jul 28;13:886319. doi: 10.3389/fimmu.2022.886319 (PMC9367496; doi:10.3389/fimmu.2022.886319)

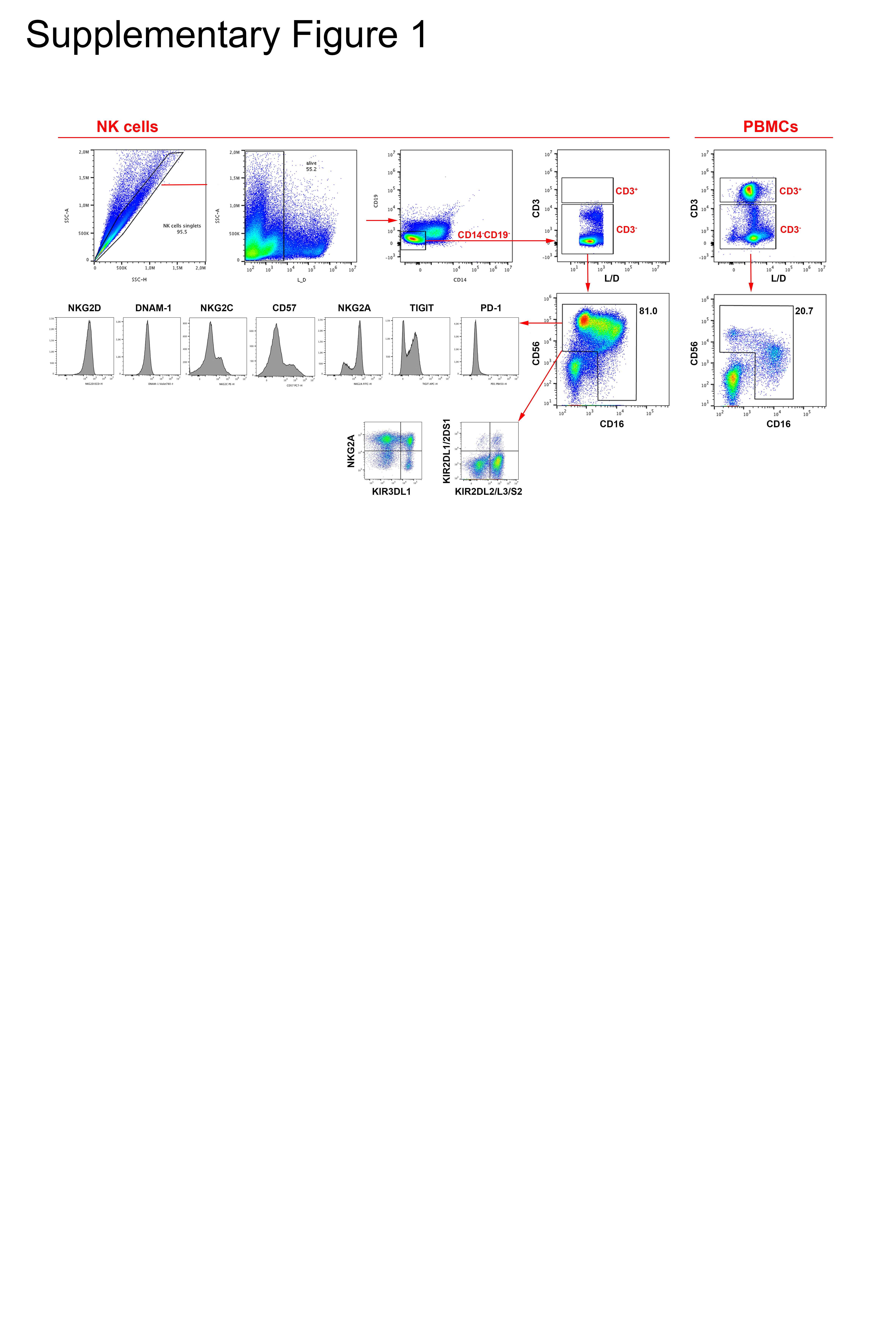

Supplement: Supplementary Figure 1 — Gating strategy used to analyse NK cell subsets by flow cytometry. In vitro-expanded and activated NK cells were checked for the expression of their specific markers at 0 (immediately after isolation from blood of healthy donors), 8, 15 days and before performing functional experiments (15-20 days) of in vitro culture in supplemented NK MACS medium. After gating singlet cells (side scatter-A versus side scatter-H), and alive cells (negative for Live/Dead staining), NK cells were identified by gating on CD14- CD19- CD3- subsets to evaluate in CD56+ CD16+ subset the expression of surface markers, as indicated. NK cells expressing NKG2A, KIR2DL1/2DS1, KIR2DL2/L3/S2 and KIR3DL1 were used for experiments. A representative staining of expanded and activated NK cells at 8 days of culture is shown. The expressions of CD3, CD56 and CD16 in cultured NK cells were compared to those in PBMCs isolated from the same healthy donors. The percentages of NK cells are shown in CD56 vs CD16 dot plots. [file Image_1.jpeg]

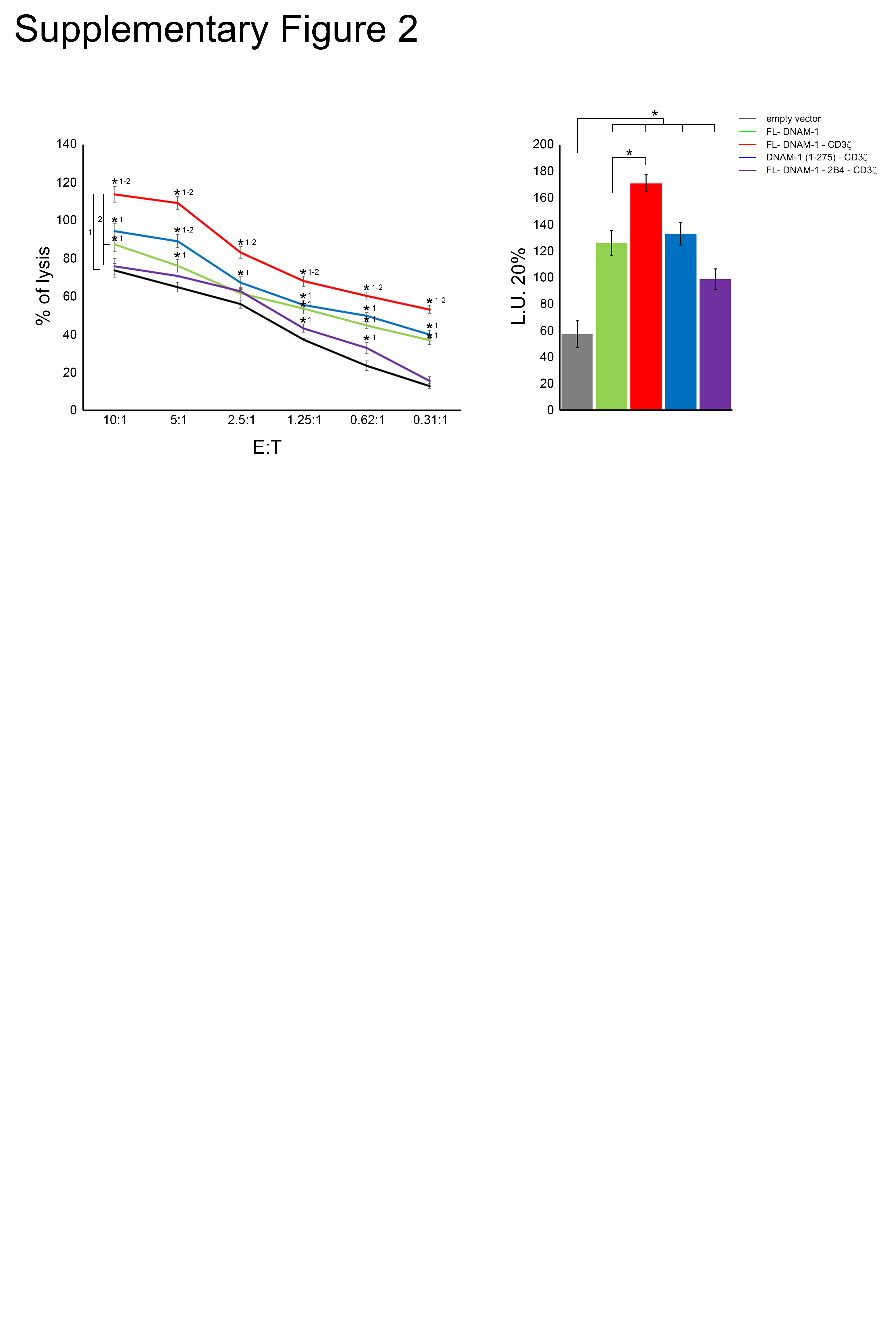

Supplement: Supplementary Figure 2 — The cytotoxic activity of DNAM-1-engineered NK cells was evaluated against K562 cells at the indicated effector:target (E:T) ratios by a standard 4-hour 51Cr-release assay. A representative experiment out of three performed is shown. The specific lysis from cytotoxic activity in triplicates were converted to L.U. 20% (right panel). The dots in the curves (left panel) and bars in the histogram (right panel) indicate the mean ± SD of triplicates; *p<0.05; p value (two-tailed non-parametric Mann-Whitney test; in the left panel, *1, empty vector versus all four DNAM-1-based vectors; *2, FL-DNAM-1 vectors versus all three DNAM-1-CD3ζ-based vectors. [file Image_2.jpeg]
