# Supplementary material for: DNAM-1-chimeric receptor-engineered NK cells, combined with Nutlin-3a, more effectively fight neuroblastoma cells in vitro: a proof-of-concept study
Source: Front Immunol. 2022 Jul 28;13:886319. doi: 10.3389/fimmu.2022.886319 (PMC9367496; doi:10.3389/fimmu.2022.886319)
Supplement: Supplementary file 3 [file DataSheet_1.docx]

**DNAM-1-chimeric receptor-engineered NK cells, combined with Nutlin-3a, more effectively fight neuroblastoma cells *in vitro*: a proof-of-concept study.**

Chiara Focaccetti^1§^, Monica Benvenuto^1§^, et al.

**Supplementary Materials and Methods**

*Amino acid sequences of DNAM-1-based constructs*

**FL-DNAM-1**

MDYPTLLLALLHVYRALCEEVLWHTSVPFAENMSLECVYPSMGILTQVEWFKIGTQQDSIAIFSPTHGMVIRKPYAERVYFLNSTMASNNMTLFFRNASEDDVGYYSCSLYTYPQGTWQKVIQVVQSDSFEAAVPSNSHIVSEPGKNVTLTCQPQMTWPVQAVRWEKIQPRQIDLLTYCNLVHGRNFTSKFPRQIVSNCSHGRWSVIVIPDVTVSDSGLYRCYLQASAGENETFVMRLTVAEGKTDNQYTLFVAGGTVLLLLFVISITTIIVIFLNRRRRRERRDLFTESWDTQKAPNNYRSPISTSQPTNQSMDDTREDIYVNYPTFSRRPKTRV

**FL-DNAM-1 + CD3ζ (52-164 aa)**

MDYPTLLLALLHVYRALCEEVLWHTSVPFAENMSLECVYPSMGILTQVEWFKIGTQQDSIAIFSPTHGMVIRKPYAERVYFLNSTMASNNMTLFFRNASEDDVGYYSCSLYTYPQGTWQKVIQVVQSDSFEAAVPSNSHIVSEPGKNVTLTCQPQMTWPVQAVRWEKIQPRQIDLLTYCNLVHGRNFTSKFPRQIVSNCSHGRWSVIVIPDVTVSDSGLYRCYLQASAGENETFVMRLTVAEGKTDNQYTLFVAGGTVLLLLFVISITTIIVIFLNRRRRRERRDLFTESWDTQKAPNNYRSPISTSQPTNQSMDDTREDIYVNYPTFSRRPKTRVRVKFSRSADAPAYQQGQNQLYNELNLGRREEYDVLDKRRGRDPEMGGKPQRRKNPQEGLYNELQKDKMAEAYSEIGMKGERRRGKGHDGLYQGLSTATKDTYDALHMQALPPR

**DNAM-1 (1-275 aa) +** **CD3ζ (52-164 aa)**

MDYPTLLLALLHVYRALCEEVLWHTSVPFAENMSLECVYPSMGILTQVEWFKIGTQQDSIAIFSPTHGMVIRKPYAERVYFLNSTMASNNMTLFFRNASEDDVGYYSCSLYTYPQGTWQKVIQVVQSDSFEAAVPSNSHIVSEPGKNVTLTCQPQMTWPVQAVRWEKIQPRQIDLLTYCNLVHGRNFTSKFPRQIVSNCSHGRWSVIVIPDVTVSDSGLYRCYLQASAGENETFVMRLTVAEGKTDNQYTLFVAGGTVLLLLFVISITTIIVIFLRVKFSRSADAPAYQQGQNQLYNELNLGRREEYDVLDKRRGRDPEMGGKPQRRKNPQEGLYNELQKDKMAEAYSEIGMKGERRRGKGHDGLYQGLSTATKDTYDALHMQALPPR

**FL-DNAM-1 + 2B4 (251-370 aa)+ CD3ζ (52-164 aa)**

MDYPTLLLALLHVYRALCEEVLWHTSVPFAENMSLECVYPSMGILTQVEWFKIGTQQDSIAIFSPTHGMVIRKPYAERVYFLNSTMASNNMTLFFRNASEDDVGYYSCSLYTYPQGTWQKVIQVVQSDSFEAAVPSNSHIVSEPGKNVTLTCQPQMTWPVQAVRWEKIQPRQIDLLTYCNLVHGRNFTSKFPRQIVSNCSHGRWSVIVIPDVTVSDSGLYRCYLQASAGENETFVMRLTVAEGKTDNQYTLFVAGGTVLLLLFVISITTIIVIFLNRRRRRERRDLFTESWDTQKAPNNYRSPISTSQPTNQSMDDTREDIYVNYPTFSRRPKTRVWRRKRKEKQSETSPKEFLTIYEDVKDLKTRRNHEQEQTFPGGGSTIYSMIQSQSSAPTSQEPAYTLYSLIQPSRKSGSRKRNHSPSFNSTIYEVIGKSQPKAQNPARLSRKELENFDVYSRVKFSRSADAPAYQQGQNQLYNELNLGRREEYDVLDKRRGRDPEMGGKPQRRKNPQEGLYNELQKDKMAEAYSEIGMKGERRRGKGHDGLYQGLSTATKDTYDALHMQALPPR
