# Supplementary material for: DNAM-1-chimeric receptor-engineered NK cells, combined with Nutlin-3a, more effectively fight neuroblastoma cells in vitro: a proof-of-concept study
Source: Front Immunol. 2022 Jul 28;13:886319. doi: 10.3389/fimmu.2022.886319 (PMC9367496; doi:10.3389/fimmu.2022.886319)
Supplement: Supplementary file 4 [file Table_1.docx]

| **Supplementary Tables**  **Supplementary Table 1**-Clinical trials in recruiting, completed and terminated status on NK cell-based immunotherapies adopting non-combination or combination approaches of chemotherapy, monoclonal antibodies (mAbs), immune checkpoint inhibitors, cytokines and adoptive transfer of NK or CAR-NK cells. | | | | | |
| --- | --- | --- | --- | --- | --- |
| **mAbs targeting NK cell-inhibitory receptors** | | | | | |
| *mAbs* | *in combination with* | *ClinicalTrial.gov identifier* | *phase* | *status* | *tumor* |
| anti-NKG2A | α-HER2, α-EGFR, α-PD-1 | NCT05162755 | I | recruiting | AST |
|  | BTK inhibitor | NCT02557516 | I-II | terminated | LL |
| anti-KIRs | - | NCT01248455 | II | terminated | MM |
|  | - | NCT00552396 | I | completed | MM |
|  | - | NCT01256073 | I | completed | AML |
|  | α-CTLA4 | NCT01750580 | I | recruiting | AST |
|  | - | NCT00999830 | II | completed | MM |
|  | α-PD-1 | NCT01714739 | I-II | completed | AST |
|  | - | NCT01222286 | II | completed | MM |
|  | - | NCT01687387 | II | completed | AML |
|  | α-CD20 | NCT02481297 | II | completed | L |
|  | - | NCT02593045 | I | completed | CTL |
|  | lenalidomide | NCT01217203 | I | completed | MM |
|  | α-PD-1, α-CTLA4 | NCT03203876 | I | completed | CTL |
| **Adoptive transfer of *ex vivo* activated NK cells in combination with cytokines, chemotherapeutics and immune checkpoint inhibitors** | | | | | |
| *NK cell origin* | *in combination with* | *ClinicalTrial.gov identifier* | *phase* | *status* | *tumor* |
| autologous | IL-2 | NCT00717184 | I | completed | MN |
| allogenic | α-PD-1 | NCT03937895 | I-II | completed | BTC |
|  | chemotherapy, α-EGFR, IL-2 | NCT02845999 | I | completed | GMC |
|  | α-PD-1/PD-L1 | NCT05395052 | I | recruiting | AST |
|  | α-PD-1/PD-L1, α-HER2, α-EGFR | NCT05069935 | I | recruiting | AST |
|  | α-GD2, chemotherapy, IL-2, GM-CSF | NCT01576692 | I | completed | NB |
|  | stem cell infusion | NCT01287104 | I | completed | L, Ly |
| haploidentical donor-derived | chemotherapy, IL-2 | NCT00698009 | II | terminated | NB |
|  | chemotherapy, α-CD22, IL-2 | NCT00941928 | II | terminated | ALL |
| haploidentical IL-15-stimulated | - | NCT01337544 | I-II | terminated | PRST |
| expressing soluble IL-15 | chemotherapy, α-PD-L1 | NCT05334329 | I | recruiting | NSCLC |
| iPSC-derived | Chemotherapy, α-PD-1, α-PD-L1, IL-2 | NCT03841110 | I | recruiting | AST, Ly |
| memory-like | IL-15 superagonist, α-CTLA-4 | NCT04290546 | I | recruiting | HNC |
| Cytokine-inducer killer cells (CIK) | α-PD-1 | NCT03987698 | II | recruiting | RCC |
|  | α-PD-1 and chemotherapy | NCT03987867 | I | recruiting | NSCLC |
|  | - | NCT01749865 | III | completed | HC |
|  | - | NCT00769106 | III | completed | HC |
|  | chemotherapy | NCT03084809 | IV | completed | CC |
| autologous CIK | - | NCT00394381 | I-II | completed | AML, My |
|  | - | NCT00815321 | II | completed | AML |
|  | chemotherapy | NCT02621333 | II | terminated | LA |
|  | chemotherapy | NCT00477035 | I | completed | HRHM |
|  | chemotherapy | NCT03002831 | II | terminated | PC |
|  | α-EGFR | NCT01871480 | II | terminated | NSCLC |
| autologous Dendritic cells-CIK | chemotherapy | NCT01395056 | - | completed | BC |
|  | chemotherapy | NCT01232062 | - | completed | BC |
| allogenic CIK | chemotherapy | NCT01392989 | II | completed | My, MyPD |
| **Adoptive transfer of CAR-NK cells** | | | | | |
| *CAR* | *in combination with* | *ClinicalTrial.gov identifier* | *phase* | *status* | *tumor* |
| α-CD19 | - | NCT05410041 | I | recruiting | BCNHL |
|  | - | NCT04887012 | I | recruiting | BCNHL |
|  | chemotherapy | NCT05020678 | I | recruiting | BCC |
|  | chemotherapy | NCT04796675 | I | recruiting | BCNHL |
|  | chemotherapy | NCT04796688 | I | recruiting | HRHM |
|  | chemotherapy | NCT05379647 | I | recruiting | BCC |
| α-CD19/CD22 | - | NCT03824964 | Early I | unknown | BCC |
| α-CD22 | - | NCT03692767 | Early I | unknown | BCC |
| α-CD33 | chemotherapy | NCT05008575 | I | recruiting | AML |
| α-CD33/CLL1 | - | NCT05215015 | Early I | recruiting | AML |
| α-BCMA | - | NCT03940833 | I-II | unknown | MM |
|  | chemotherapy | NCT05008536 | Early I | recruiting | MM |
|  | chemotherapy, α-CD38 | NCT05182073 | I | recruiting | MM |
| α-PSMA | - | NCT03692663 | Early I | unknown | PC |
| α-ROBO1 | - | NCT03940820 | I-II | unknown | AST |
|  | - | NCT03941457 | I-II | unknown | PC |
|  | - | NCT03931720 | I-II | unknown | AST |
| PD-L1 | IL-15RαFc superagonist, α-PD-1 | NCT04847466 | II | recruiting | GMC, HNC |
| α-5T4 | - | NCT05194709 | I | recruiting | AST |
|  | - | NCT05137275 | Early I | recruiting | AST |
| α-HER2 | - | NCT03383978 | I | recruiting | G |
| Claudin6 targeting | - | NCT05410717 | I-II | recruiting | AST |
| NKG2D | - | NCT05213195 | I | recruiting | MCC |
|  | - | NCT05247957 | I | recruiting | AML |
| NKG2D-ACE2 | - | NCT04324996 | I-II | recruiting | COVID-19 |
| NKG2D ligand targeting | chemotherapy | NCT04623944 | I | recruiting | AML |
|  | IL-2 | NCT03415100 | I | unknown | AST |
| **Supplementary Table 2**-Completed and terminated clinical trials on the use of MDM-2 targeting drugs in combination or not with chemotherapeutics and immune checkpoint inhibitors. | | | | | |
| **MDM2-targeting drugs** | | | | | |
| *Drug* | *in combination with* | *ClinicalTrial.gov identifier* | *phase* | *status* | *tumor* |
| RO5045337 | - | NCT01677780 | I | completed | LL |
|  | - | NCT01143740 | I | completed | LS |
|  | chemotherapy | NCT01605526 | I | completed | LL, S |
| RO6839921 | - | NCT02098967 | I | completed | LL |
| Idasanutlin | - | NCT03362723 | I | completed | AST |
|  | MEK inhibitor, α-PD-L1 | NCT03566485 | I-II | terminated | BC |
| HDM201 | - | NCT02143635 | I | completed | AST |
|  | CDK inhibitor | NCT02343172 | I | completed | LS |
| APG-115 | - | NCT02935907 | I | completed | AST, Ly |
| ALRN-6924 | - | NCT02264613 | I-II | completed | AST, Ly |

Abbreviation: ALL, Acute Lymphoblastic Leukemia; AML, Acute Myeloid Leukemia; AST, Advanced Solid Tumors; BC, Breast Cancer; BCC, B-cell cancers; BCNHL, B-cell non-Hodgkin’s lymphoma; BTC, Biliary Tract cancer; CC, Colorectal cancer; Coronavirus disease 2019, COVID-19; CTL, Cutaneous T-cell lymphomas; G, Glioblastoma; GMC, Gastrointestinal Metastatic cancer; HC, Hepatocellular carcinoma; HNC, Head and Neck cancer; HRHM, High-risk Hematologic Malignancies; L, Leukemia; LA, Lung Adenocarcinoma; LL, Lymphocytic Leukemia; LS, Liposarcoma; Ly, Lymphoma; MCC, Metastatic Colorectal cancer; MM, Multiple Mieloma; MN, Metastatic Nasopharyngeal; My, Myelodysplasia; MyPD, Myeloproliferative disorders; NB, Neuroblastoma; NSCLC, Non-Small Cell Lung Carcinoma; PC, Pancreatic cancer; PRST, Pediatric refractory solid tumors; RCC, Renal cell carcinoma; S, Sarcoma.
